# Public Health Nutrition: The Accord of Dietitian Providers in Managing Medicare Chronic Care Outpatients in Australia

**DOI:** 10.3390/ijerph7041841

**Published:** 2010-04-20

**Authors:** Robyn P. Cant

**Affiliations:** Research Fellow, School of Nursing and Midwifery, Monash University, Churchill, Victoria 3842, Australia; E-Mail: robyn.cant@med.monash.edu.au; Tel.: +61-9902-6426; Fax: +61-9902-6527

**Keywords:** allied health, Australia, dietetics, Medicare, Medicare chronic disease management, primary care

## Abstract

Medicare Australia: Chronic Disease Management program subsidizes allied health consultations for eligible outpatients with chronic disease or complex needs. In an evaluation study, private practice dietitians (n = 9) were interviewed to explore their patient management strategies including consultation time-allocation and fees. Time allocation was fee-based. Short first consultations were seen as meeting patients’ needs for low-cost services but were regarded by dietitians as ineffective, however longer initial consultations increased cost to patients. No strategy in use was optimal. There is a need for change in Medicare policy to meet the needs of both dietitians and patients in achieving the behaviour change goals of patients.

## 1. Introduction

The Australian government has an increasing focus on public health approaches to the health of the community with the objective of providing ‘more preventive care for older Australians’, and ‘improving co-ordination of care for people with chronic conditions and complex care needs’ [1]. The Medicare Chronic Disease Management (CDM) Allied Health Program was introduced nationwide in 2004 to aid this objective [2].

As in other countries, an aging population with increasing incidence of chronic diseases such as cardiac disease, diabetes, or hypertension, led to this policy change. In Australia, chronic medical conditions are responsible for over 80% of the overall burden of disease and injury [3]. This adds to already high health care costs and raises a dilemma in relation to public health policy: how to reverse this trend?

**Figure 1.**
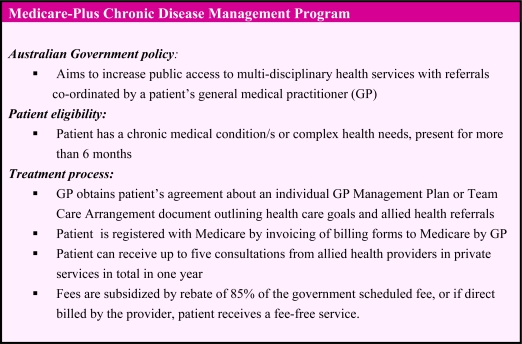
Summary of Medicare-Plus CDM program. Source: Enhanced Primary Care Program: Chronic Disease Management Medicare Items [2].

The Enhanced Primary Care (EPC) program that was commenced in 1999 aimed to engage general medical practitioners (GPs) in the coordination of the medical care of their patients using subsidized medical services. These are funded under the national taxation-based health insurance: Medicare. The Medicare-Plus Chronic Disease Management (CDM) Allied Health Program aims to positively impact the health of patients with chronic disease or complex health needs [2]. This allows eligible patients access to various allied health professional services in private clinics at subsidized fee rates. The program supplements public services in hospitals, hospital clinics and community-based health and medical services such as multidisciplinary community health services. A summary of the program is given in Figure 1 and further details are described below.

In order to be eligible for access to Medicare CDM, a patient’s GP is required to prepare an individual written care plan. This is a GP Management Plan (GPMP) or a Team Care Arrangement (TCA). These processes are complex: details of GP, dietetic and other allied health services funded under Medicare are given on the website of Medicare Australia (http://www.medicareaustralia.org). For a GPMP, a GP collaborates with a patient; assesses the patient, agrees their goals for health improvement, identifies actions to be taken by patient, identifies treatment/ongoing services to be provided, and documents the plan. A TCA requires GPs to coordinate team-based care for a patient with a chronic condition and complex, multi-disciplinary care needs and this should involve at least three professionals in planning [2].

Patients agree to the care plan and the suggested allied health consultations and receive a copy of their plan with the treatment goals. Allied health interventions aim to engage patients in learning, including of new skills and this concurs with chronic disease management principles that show that increase in the self-management skills of patients is associated with improvements in their health outcomes [4]. Each eligible patient is entitled to up to five subsidized consultations from approved allied health services in private clinics each year [2]. In the case of a patient with diabetes, this potentially includes, diabetes educator, dietitian and/or podiatrist so that these consultations must be shared between provider professions. Patients have renewed entitlements annually.

As chronic disease risk is commonly related to dietary choices [3,5], dietetics referrals may relate to diagnoses such as diabetes, or a cardiac or other condition. A GP or practice nurse in consultation with the patient sets dietary goals for patients to achieve with a dietitian’s counselling assistance. Each consultation must be at least 20 minutes duration face to face with a practitioner, and the patient can receive a rebate of 85% of the scheduled fee when the scheduled fee is charged. The scheduled fee for both initial and follow-up dietetics consultations was AU$58.85 at November 2009, and the rebated remuneration for providers per visit AU$50.05 [6].

Dietetics is the third most utilized of 14 allied heath provider groups, including physiotherapy, podiatry/chiropody, chiropractic, speech pathology, exercise physiology, diabetes education [7]. The number of dietetics consultations conducted nationally has been rising every year [8]. This program offers substantial business opportunity to private practices to increase their workloads in clinic environments which include multidisciplinary practices with a GP or without a GP, sole practices, or group practices comprising a group of dietitians [8].

Published evaluation of Medicare CDM policy implementation is lacking and no data is available about the health outcomes of chronic disease patients. However, several studies have explored providers’ perceptions of the program’s impact. Foster *et al.* reported results of a qualitative study of five professions in Queensland (exercise physiologists, occupational therapists, physiotherapists, social workers and speech pathologists) in 2006 [9]. They reported care provision was affected by the small remuneration paid. Practitioners who were constrained by the prescribed consultation or repeat visit limits condensed their time management in caring for a patient, resulting in an ‘abbreviated care’ model. There was a need for resolution of some issues, including health practitioners’ obligation to provide an adequate professional service whilst ensuring the financial viability of the business practice. This concurred with the views of 15 dietetics providers who were interviewed in the first twelve months of the program in 2005. The initial, or first, consultation time they allowed a patient (which they perceived as limited by the set remuneration) was largely inadequate time to help patients to achieve their dietary goals [10].

A national survey of Australian dietitians in private practice (n = 356; 47%) by the author in 2007 reported a need to review a number of areas of the Medicare-CDM policy to prompt an increase in dietitians’ endorsement of the program [11]. Both the funded initial consultation time and the available consultation frequency were, in the main, seen as inadequate to allow dietitians to assist patients to achieve the dietary goals set by GPs. Dietitians needed more time with a patient to assess, educate, set dietary goals and to monitor progress- as confirmed in dietetics practice principles for chronic disease management [12]. Income from the Medicare fee rebate was said to be less than their usual fee scale.

The quantitative results of the survey of dietitians were reported earlier [11]. Due to the Medicare policy constraints, dietitians applied various consultation strategies in developing their service for Medicare- eligible patients. These adaptations of practice have broad implications for both patients and practitioners in the delivery of CDM dietetics services. This paper aims to explore the care models of Medicare CDM service provision that dietitians adopt in order to comply with the policy guidelines and yet maintain their business plan and the economic viability of private clinics. A qualitative approach will be used.

## 2. Methods

Interview technique was utilized as the primary data source [13]. A purposive sample of 20 dietitians who were Medicare providers was selected from those who consented to be interviewed when responding to the national survey mentioned above; nine were then interviewed. Sample selection was made on the basis of capturing the variation apparent in practice environments [14]. This was firstly, sampling both low use Medicare providers of <10 referrals per month and high use (>26 referrals per month), and then the clinic category in which they were employed (sole practice or group practice) and providers in the major dietetic provider states (NSW, Victoria, Queensland, South Australia, West Australia). Second, both those who were satisfied with the Medicare program and those who were not satisfied were purposely selected. Each interview time was pre-arranged and if a dietitian was not readily available for interview within the next week, then the next on the list was contacted for interview.

A single experienced researcher (RC) interviewed the dietitians by telephone over 25 to 40 minutes each in 2008 using a semi-structured interview technique. An interview schedule built on data from the national survey was used to initiate discussion. This asked about matters such as the time given to consultations, perceptions of patients’ needs and detail of reporting methods. Questions were intended to clarify responses or comments these same individuals had made in their returned surveys and/or to further focus the themes identified in the overall survey results. Each participant had already responded to a questionnaire and given their identity and practice details so previous responses were summarized for the interviewer for each participant on a running sheet prior to interview. Some questions were leading in order to gain further perspectives on overall responses. Examples of questions are given in Table 1.

Subsequent questions aimed to further clarify an issue pertaining the particular category of practice or the management process used. For example, a dietitian co-located with a GP might manage referrals, billing and communications quite differently to those in sole practices. Furthermore, as the interview process was iterative, questions were altered to probe for more detail depending upon the responses of a previous interviewee, as expected according to in-depth interviewing technique [13].

The interviews were audio-recorded with each participant’s consent. Interviews were subsequently ceased after analysis of nine interviews as no new topics were raised and it was thought that a ‘saturation’ of topics was evident [13]. In part, this was due to questionnaire data having already provided much detail of customary practice, including narratives given in the invited open-ended comments. The audiotapes were studied with notation of the narrative themes to develop a matrix of various responses and this analysis continued simultaneously with interviewing. A constant comparative technique was used to compare similarities or differences between the views of dietitians from different employment environments, practices, and states of employment [15]. The findings were subsequently compared with themes from the questionnaire results, including those from open-ended comments- in a process of triangulation [13].

The study was approved by the human ethics committee of the sponsoring university. No financial or other inducement was offered to participants for their contribution to the study.

## 3. Findings

The findings from analysis of the interview data are presented in order to build a logical chain of evidence about practice conditions. Some quotations from the narratives were selected and are presented as indicative of the views of one sector of dietetics practice.

### 3.1. Participant Characteristics

The nine female dietitians interviewed were employed in a wide range of clinic businesses including (i) sole practice, (ii) multi-professional allied health practice (iii) group dietetics practice (iv) group practice with GP, in both metropolitan and country regions. This encompassed the states with the largest number of dietetics Medicare transactions: NSW, Victoria, Queensland, South Australia, and West Australia. Several were located with a GP referrer in the same building, and all received their CDM referrals by letter. Their hours of work in private practice ranged from seven hours per week to full-time. They first qualified for dietetics between 1972 and 2006, although most were experienced with at least five years of experience. Some were satisfied with the Medicare CDM program and some were dissatisfied, and thus were able to give diverse views about the impact of the program.

### 3.2. Initial Consultation Strategies

The Medicare fee rebate was thought by all to fund a short consultation of 20–30 minutes, given the cost of extra time for writing reports to doctors and cost of office overheads and administration. Four main strategies were applied to first consultation arrangements in an endeavor to meet CDM patients’ needs for nutrition education whilst also complying with the policy guidelines and their business principles. There was no overall pattern of consultation management strategy found by any particular employment environment: all differed in their decisions. No major new themes were identified, as each dietitian voiced issues that were apparent in the earlier survey, but through the interview data, enabled more detailed perspectives and filled in information gaps.

Much of the interview discussion applied to a first consultation, which was of principal concern to all the dietitians due the high frequency of occurrence of these referrals and the perceived high degree of difficulty of the counselling for some patients. Dietitians might see a patient once or twice for a consultation. They made a choice of these initial consultation arrangements:

- to limit all consultations to 20–30 minutes
- to deliver a longer initial consultation of 45 to 60 minutes and charge patients a fee gap
- to deliver an initial consultation longer than 20–30 minutes at no extra fee
- to choose not to accept (or to accept few) CDM patients.

The second option involving an increase in fees was the most common, thus adding to a patient’s out-of-pocket costs. Interviews provided detailed information about how the consultations were managed.

#### (a) The 20-minute consultation

Several dietitians had experience of conducting short initial consultations, depending on the clinic environment, although this was much more common for review consultations. One dietitian routinely scheduled 20-minute initial and review consultations for all Medicare CDM patients, working within the current national fee scale for bulk-billed consultations. This fulltime employee in a group dietetic practice was a high use provider, seeing 50–100 CDM patients per month. Receptionists handled administration and the bulk- billing of invoices to Medicare, which meant that patients were not required to pay any fee contribution for their consultation. She stated that the allowed number of visits was sometimes adequate but “*most often it is not*”, and patients were often asked to return for additional follow-up consultations for which they themselves would pay:

“Because I am not fitting as much in my initial [consultation], then there is always more that we can go through. And so these people are happy to come back for a review, and then er pay for a review, and some people are happy to continue for an ongoing period of time. …For some people two, three, four, five visits is adequate, but usually up to ten.”

The 20-minute process was in contrast to her other fee-paying patients for whom a first consultation was 40 minutes and the fee was correspondingly doubled. Consultation report letter-writing to GP referrers required additional time, although this was greatly facilitated by use of entries in case notes using electronic software which was shared by GP and other allied health providers. For other referrals from outside this clinic, consultation report letters were written.

#### (b) Longer consultation, charge patients a fee gap

Several dietitians saw the need to provide longer consultations of up to one hour at the first visit. For example, a medium volume provider working in several private clinics in a capital city, both located within a GP clinic and in a multidisciplinary practice without a GP, stated:

“the rebate for dietitians needs to be higher- we can’t do our job properly in 30 minutes!”

Another dietitian explained why this was so:

*“Oh, my initial consult needs to be about 45 to 50 minutes,* *usually* *at least an hour. You see, the whole thing is- creating rapport with these patients, taking a* *really* *good history of where they have been at. And then, when you are working with them, to try and identify the places that they are at, you know? What they are happy to work with [to try and change]. And that takes a good part of an hour!”*

A dietitian working in a large provincial town with up to 25 CDM new patients per month justified this process:

*“I treat them like any other client that comes and sees me. And I feel in a way, you know, that these clients need* *more* *time, they need* *more* *attention, because mostly they’ve got a heap- a heap of chronic conditions, and they need* *a lot more* *management, a lot more counselling. So- basically, everyone I see in the initial consult, it’s an hour. It’s that same for everybody. I don’t charge them less because I am putting in just as much effort. In fact, more! You know? …It is $80, and I tell the people this when they book in.”*

However, introduction of higher payments without direct billing to Medicare meant that patients paid a fee gap and did not receive a low-cost service. In the above case, a patient would pay AU$30.05 from their pocket. As some patients were under the impression that they could receive a ‘fee-free’ service upon referral, one effect of this was that some doctors referred to other lower cost practices. Dietitians were asked about the impact of higher fees for longer consultations. A low volume Medicare provider (<10 referrals per month) in sole suburban practice commented when asked about feedback from patients about how much they have to pay in the gap payment:

*“Most are* *fine. I mean, they understand generally that there is going to be a gap. There are some who, once they realise there is going to be a gap, then they won’t make an appointment. Um- so for those, obviously, I mean, it’s definitely an issue. And it will impact on whether they have any input from* *anyone.”*

There were various comments about patients’ ability to continue attending the clinic according to their ability to pay for follow-up consultations, even though this involved shorter and cheaper consultations. Patients possessing private health insurance were thought to more often attend for review appointments.

#### (c) Longer consultation at no extra fee

Several dietitians echoed the need to provide CDM patients with longer initial appointments of 45 or 60 minutes, whilst not increasing their fees above the scheduled fee. A dietitian working in a suburban multidisciplinary practice stated that the fee structure was constrained by doctors who said they would only refer patients if the patients’ fees were directly bulk-billed.

*“We bulk bill all Medicare services. However, we take a considerable loss of $42 on every new patient we bulk-bill. This is a huge stretch of professional ethics: financial viability* versus *adequate dietetic care.”*

Another dietitian working in a group dietetic practice quoted business competition as the reason for their fee scale.

“…unsure of future bulk-billing- we will do this with a one-off visit of $50 as we are losing custom to dietitians who are- even if we know they cannot be doing an adequate job with the time constraints. The quality of care must not be compromised...”

There were inferences about the differences in fees paid to GPs for preparing a patient’s care plan and perceptions of a low payment made to dietitians in carrying out the tasks of patient education. A GP will receive more than twice the fee rebate paid to a dietitian for one consultation for their preparation of a care plan for a patient. A dietitian, in commenting about Medicare CDM program stated:

“The concept is good but it is benefiting the GP’s pocket more than anything else.”

Follow-up or review dietetic appointments were commonly shorter, they fitted the dietitians’ time schedule and patients were often charged the scheduled fee. Thus, these follow-up consultations were regarded as less onerous.

#### (d) Limit CDM patients, or not accept them

Part of the patient referral process is that medical clinics should seek the approval of dietitians for the ‘care plan’ of a patient before sending the referral to a dietetics clinic. However, this did not work effectively. Dietitians were not always asked to have input into the plan. Some dietitians were ambivalent about the number of CDM patient referrals they would accept and interview discussions revealed two main reasons why dietitians might place these limits. Dietitians may have waiting lists for patients and not be intending to increase the overall number of referred patients, and/or they may wish to limit the proportion of CDM patients in their practice because of the relative difference in the remuneration and perceptions of greater reward from other paying private patients. This refers not only to the difference in fees. Dietitians also commented on expectations of professional reward they might receive due to assisting the patients. This was interpreted as applying through their main objective: “*giving a quality service*” to patients. This theme recurred in interviews and conversely, concerns about short initial consultations were linked with dialogues about not meeting the needs of patients. CDM patient referrals were often seen by dietitians who voiced these attitudes as complex, burdensome, unprofitable, and also costly in terms of the extra administration time and the required report-writing.

### 3.3. Administrative Costs

While several dietitians received assistance from administrative staff who facilitated appointments, copying, billing and so on, some providers managed these tasks alone. Workforce statistics indicate that a number of providers work sessional or part-time hours in sole positions and these may be less likely to receive administrative help. These factors may negatively influence acceptance of the program, for it was clear that dietitians preferred patient contact to dealing with administrative “*red tape*”. Administrative costs were thus both finite and also induced.

Prominent in the discussions were issues of the time required for mandatory report-writing under the Medicare CDM policy. It was clear that dietitians regarded these tasks as costly because they were unpaid additions to the care of patients in an environment where time had a price. A dietitian commented:

“I am not getting paid for the time I take to write reports”.

Another dietitian commented

*“I write my reports after; I have a writing day when I do my reports, my admin. …Its all personalized. And that’s where I have templates…and it takes* *time.”*

Dietitians are required to provide written reports to GPs on completion of the initial CDM consultation and the final visit, often requiring two letters after two visits. One reason for dietitians’ reluctance may be the use of paper-based or open text letters rather then more structured letters that can be populated with patient information more easily and quickly. A letter was reported to take “*five to ten minutes*”. Two interviewed dietitians worked in clinics that shared electronic medical records between doctor and dietitian via shared computer software and this appeared to facilitate the communication:

“We’re all computerized and um, we’ve got templates. So I just put in the individual details into the template and then send it. …Its fantastic!”

This illustrates the potential for cost containment if electronic systems are available for reporting back to referring GPs. All the operations of the dietetics clinics were reportedly constrained by economic factors that they related as being vital to the viability of the clinic business.

## 4. Discussion

These findings illustrate some of the barriers and some facilitating factors regarding dietitians’ perceptions of what constitutes effective management of patients with chronic disease referred by GPs under Medicare-Plus. Although patients funded under the program offered considerable new business to these healthcare operations, a consistent underlying message was a competing need to conduct viable business operations through receipt of adequate levels of remuneration for completed work. As the Medicare MBS schedule offered low remuneration [6] compared with dietitians’ usual fee structure, the views of dietitians were that patient care could be compromised.

### 4.1. Fee Structures Impose Practice

Fee income appeared to pre-empt the consultation time-allocation and time management decisions of dietitians in the private practices. In some cases, time management decisions were made by the individual dietitian and in others by an employer. This was so in the case of a group practice comprising salaried dietitians. Alternatively, fees were indirectly imposed by external forces. There were GP referrers who might set price limitations and voice their unwillingness to refer patients if this demand was unmet, and market forces whereby practices that might compete for patients voluntarily set similar fee structures.

As a result of decisions to provide short initial consultations, it could be inferred that Medicare CDM patients were receiving a lower standard of care than full-fee paying patients. Less time for counselling demanded that dietitians compress and shorten usual counselling content. This difference was well recognized by interviewed dietitians and was illustrated by one who reported conducting 20-minute initial consultations for CDM bulk-billed patients and 40-minute consultations for all others. The ‘others’ were paying more than twice the CDM consultation fee. These decisions were reported as being forced by the MBS fee structure which applied to the allied health providers, and also dietetics, and illustrate that discounted rates may result in patients being given a discounted level of service. Foster *et al.* found in their study of Medicare providers in Queensland that remuneration restricted the conditions of allied health practice and imposed a less than optimum allied health service [9]. This allied health provider group comprised exercise physiologists, occupational therapists, physiotherapists, social workers and speech pathologists. Furthermore, as the number of allied health consultations is limited to five in any year for any and all professions for patients who might need multidisciplinary care [1], there is little opportunity for repeat consultations even though these patients require- by nature of the program- continuing care for their chronic disease. It would seem important that the health outcomes of referred CDM patients are evaluated to determine the impact of these interventions and identify any positive effect of the governments’ allied health investment upon patients’ behaviour change and healthcare outcomes.

Acceptability of the 20-minute consultation among private practice dietitians is thought to be low. Interviewed dietitians as well as those in the national survey justified longer consultations, stating that more time was needed to achieve their usual process of nutrition education, a response that was prominent when dietitians were discussing the initial consultation. This concurs with evidence-based practice guidelines for chronic disease diagnoses such as diabetes or obesity where more comprehensive dietetics interventions are recommended to help change an individual’s dietary behaviours [16,17]. Best practices focus on patient centredness and a dietetic process which includes a counselling process of the steps: assessment, education, goal setting and monitoring of outcomes. This requires time to implement and revision, also reinforcement of new information and a review of outcomes whilst a dietitian is face to face with a patient. These principles are also central to the Medicare CDM policy which supports patient self-management and development of their learning to enhance understanding of their medical condition [2]. Furthermore, patients referred under Medicare CDM have *chronic disease* or *complex conditions* and dietary risk factors that make lifestyle interventions more complex. It is unlikely that dietetic change can be achieved almost instantaneously. In the earlier national study, fees seemed to relate to time given for the first consultation and, as only six percent of dietitians charged the scheduled fee [11], this suggests that dietitians found most initial consultations were necessarily longer than 20 minutes. There was greater acceptance of a shorter follow-up consultation.

### 4.2. Ethical Obligation to Patients

There was particular dissonance between the moral obligations of the dietetic practitioner to provide a professional level of performance: ‘*good quality*’ patient care, *versus* their comments about briefer interventions. Some dietitians chose to conduct practice which matched their professionalism objective by providing a standard consultation despite the extra part of this time not being remunerated. A dietitian who operated a group dietetics private practice intentionally asked staff to allocate longer time to patients’ education free of additional charge and noted that the income losses could not be permanently sustained. This ethical issue was voiced by other allied health professionals working with CDM patients in Queensland [9]. These providers described a need to reconcile “a sense of moral obligation to assist patients who could not otherwise afford to pay for allied heath services on the one hand, and on the other, the values of private enterprise that compelled them to ensure the financial viability of their practices [9; p. 330]. It is therefore unsurprising that some dietitians preferred to limit or else not accept CDM patients and similar reasons are suggested as underlying their decisions. In a national survey of 356 private practice dietitians (who were 47% of all providers), six percent (n = 14) chose not to enroll as providers for Medicare and of the accredited Medicare providers, four percent (n = 13) did not manage Medicare CDM patients [11]. No solution was found in the interviews to this paradox except for review and change of the policy to better meet the needs of both dietitians and patients in the provision of nutrition education and counselling.

### 4.3. No Optimum Model of Counselling Practice

It appeared that none of the consultation strategies used by dietitians provided a nutrition intervention model which was optimal for both dietitian and patient. There was no accord of dietitians in use of any common consultation strategy for managing CDM patients’ referrals. Short first consultations were seen as meeting patients’ needs for low cost services but were regarded as ineffective by dietitians. Longer initial consultations were preferred by dietitians, but were costly in fees for patients to pay from their own pocket or else loss-making for dietitians. Extra time required for administration of the program and reporting-writing was consistently mentioned and regarded negatively because it was not funded. The initial consultation time was also contentious because a dietitian might see a patient only once and therefore felt the need to fully complete their dietary education at the time [11]. The care models of Medicare CDM service provision that dietitians adopted did not meet their expectations for patient care whilst being viable in terms of economics for their businesses. A solution appears to be the re-structure of Medicare CDM policy to allow dietitians one longer consultation and multiple short consultations for achievement of their nutrition education and counselling tasks, assisting patients to reach the dietary goals set by them and their GP.

In the future it is expected that CDM referrals will continue to increase as they have in recent years [7,8] as more GPs become familiar with the treatment options for their chronic care patients. This will place referral decisions under more scrutiny and requires investigations into optimum models of practice. Podiatry and physiotherapy have experienced exponential growth in service provision to the extent they now provide almost 75% of all Medicare CDM services billed [7]. It may be that these allied health services are both necessary and fit naturally into the program because they are ‘treatment’ oriented and offer a hands-on patient service, in contrast to more elaborate counselling provided by dietitians. However, the limit of five allied health consultations annually presents GPs with a need to address the competing needs and priorities of a patient’s CDM referrals. Many patients have co-morbidities and require assessment and follow-up by a range of health professional providers in the CDM program, so that these referrals have to be juxtapositioned between multiple professions. Thus, policy constraints may presently be widespread.

The intention of the Australian Government to provide better care for people with chronic medical conditions via Medicare allied health dietetic interventions has not realized low cost care for this group of patients. Although patients might be agreeable to co-payments for medical services [18], in this case patients in a lower socio-economic group with greatest burden of disease [19] have less opportunity to receive dietetics services due to the service cost. Research is needed to understand what impact the Medicare CDM program interventions have had not only upon dietetics chronic care patients, but also those of the other allied health provider professions. Current annual government investment of around AU$8 million for 164,000 dietetics services to individuals [7] should be justified by evaluations of the quality of care, including examination of patient outcomes.

## 5. Conclusions

There was no accord between dietitian providers for Medicare-Plus CDM who employed various strategies for managing patient consultations. None of their time-allocation strategies provided optimal consultations for both dietitian and patient at the first visit. There was greater acceptance of policy constraints via short follow-up consultations. There is a need for review of Medicare CDM policy for dietetics providers to align fees with longer initial consultation time for a counselling type service and to restructure the fee schedule. If this was the case, patients might access low-cost preventive dietetics services that are more attuned to both patients’ and providers’ needs. Further research is necessary to determine best practice in how to achieve the behaviour change goals of chronic care patients under the Medicare-Plus CDM dietetics program and to understand the operations of other allied health providers.

## Figures and Tables

**Table 1.** Examples of initiating interview questions.

**Issue**	**Question**
**Consultation length**	Dietitians state that the current rebate did not allow sufficient time for the first consultation. Should there be a fee structure for a short and a long consultation? Would a 50-minute and a 30-minute consultation if allowable be adequate for your services?
**Reporting**	How much time would you devote to patient education *versus* writing up a report?
**Administration**	How much time does billing take?
**Best practice**	How well you think the current program meets best practice for chronic care management?
**Suggested changes**	Do you have any other suggestions about how the current program could meet patients’ needs for nutrition education?
